# Prevalence of IgM and IgG antibodies to SARS-CoV-2 in health care workers at a tertiary care New York hospital during the Spring COVID-19 surge

**DOI:** 10.1186/s13741-021-00177-5

**Published:** 2021-03-02

**Authors:** Lillian R. Talbot, Jamie L. Romeiser, Eric D. Spitzer, Tong J. Gan, Sunitha M. Singh, Bettina C. Fries, Elliott Bennett-Guerrero

**Affiliations:** 1 Renaissance Stony Brook School of Medicine, Stony Brook, USA; 2 U.S. Department of Veterans Affairs - Northport VA Medical Center, Northport, NY USA

**Keywords:** Health care worker, Intensivist, COVID-19, SARS-CoV-2, Humoral immunity

## Abstract

**Background:**

Health care workers (HCW) such as anesthesiologists, surgeons, and intensivists face high rates of exposure to SARS-CoV-2 through direct contact with COVID-19 patients. While there are initial reports of the prevalence of COVID-19 antibodies among the general population, there are few reports comparing the seroprevalence of IgM/IgG COVID-19 antibodies in HCW of different exposure levels as well as different HCW professions.

**Methods:**

A convenience sample of health care workers provided blood for COVID-19 antibody testing and a review of medical history and work exposure for correlative analyses.

**Results:**

Overall, 474 HCW were enrolled in April 2020 including 102 front-line physicians (e.g., anesthesiologists, surgeons, intensivists, emergency medicine), 91 other physicians, 135 nurses, 134 other clinical staff, and 12 non-clinical HCW. The prevalence of IgM or IgG antibodies to SARS-CoV-2 was 16.9% (95% CI 13.6–20.6) (80/474). The proportion of positive antibodies in the PCR + group was significantly higher than health care workers without symptoms (84.6% [95% CI 54.6–98.1] vs. 12.3% [95% CI 8.5–17.2], *p* < 0.001). No significant differences in proportions of COVID-19 antibodies were observed among the different exposure groups (e.g., high vs minimal/no exposure) and among the different HCW professionals.

**Conclusions:**

Despite exposure to COVID-19 patients, the prevalence of antibodies in our HCW was similar to what has been reported for the general population of New York State (14%) and for another New York HCW cohort (13.7%). Health care workers with higher exposure rates were not more likely to have been infected with COVID-19. Therefore, these data suggest that infection of HCW may result from exposure in the community rather than at work.

**Trial registration:**

This investigator-initiated study was observational; therefore, no registration was required. Not applicable.

**Supplementary Information:**

The online version contains supplementary material available at 10.1186/s13741-021-00177-5.

## Introduction

The New York Metropolitan area reported a very high number of COVID-19 infections and deaths in the initial “surge” in the USA. The first COVID-19 patients to present to Stony Brook University Hospital in Suffolk County, New York, on 8 March 2020 reported their symptoms began on 1 March 2020. Suffolk County is located 40 miles east of Manhattan and at that time reported the fifth highest number of COVID-19 cases in New York State and the second highest number of cases outside of New York City.

During the early phase of the COVID-19 pandemic, before transmission dynamics were established and adequate personal protective equipment (PPE) mandated, health care workers (HCW) were particularly vulnerable to COVID-19 infection. Health care workers, such as anesthesiologists, surgeons, and intensivists, face high rates of exposure to SARS-CoV-2 (Heinzerling et al., 2020; Team, 2020; Zhan et al., 2020; Remuzzi & Remuzzi, 2020). While there are initial reports of the prevalence of COVID-19 antibodies among the general population, there are few reports about the prevalence and association with exposure and symptoms among health care workers especially during the initial Spring “surge” in the New York/Northeast area. Therefore, the objectives of this study were (1) to assess the seroprevalence of COVID-19 antibodies in a cohort of health care workers at a New York tertiary care hospital during the “surge” (April 2020) and (2) to compare the seroprevalence between different health care worker professions and determine if individuals with higher self-reported at-work exposure to COVID-19 were more likely to have a detectable antibody response than those with lower self-reported exposure.

## Methods

Approval was obtained on 25 March 2020 from Stony Brook University’s Institutional Review Board (IRB) to study the seroprevalence of IgM and IgG antibodies in health care workers on site at Stony Brook University Hospital. The study was broadcasted via email and through respective supervisors. Health care workers were enrolled over a period of 17 days (2–18 April 2020). After obtaining written informed consent, participants were interviewed and asked to report past medical history, demographics, work exposure to COVID-19, and results from any COVID-19 polymerase chain reaction (PCR) tests performed. Participants were required to be free from any active symptoms of illness at the time of study entry.

COVID-19 antibodies were assessed using the Chembio Nucelocapsid DPP COVID-19 Assay according to the manufacturer’s instructions (Chembio Diagnostics, Inc located in Medford, New York, USA). In brief, a blood sample was obtained from a finger stick. Using a plastic loop, 10 μL of whole blood was transferred from the puncture site into a reaction tube with 5 drops of assay buffer. One hundred microliters of this buffer and blood sample mixture were transferred with a calibrated micropipette into the Chembio well and allowed to incubate for 5 min. Nine drops of assay buffer were loaded into the second well. Capillary flow washes antigen-bound antibodies over the printed antigen IgM and IgG lines. An internal process control appears in the far right of the lane approximately 5 min after the assay buffer has been added to confirm the test was done properly. In positive samples, a visible second band (due to binding of colloidal gold-labeled conjugate antibodies) will appear to the left of the control band in either or both lanes within 10 min. The test cartridge is inserted into the reader where the density of the left-most band is determined and displayed on the reader’s digital display. Based on the manufacturer’s instructions at the time of testing, reflectance light units (RLU) of > 25 were reported as “reactive.” This assay had Emergency Use Authorization (EUA) from the US Food and Drug Administration (FDA) at the time of our study. After the study was completed, the Food and Drug Administration revoked the Emergency Use Authorization due to concerns about inferior specificity and ability to detect COVID-19 antibodies at low titer, relative to other EUA-approved COVID-19 serological assays (FDA, 2020). After the conclusion of our study, our Hospital’s Clinical Laboratory conducted an independent comparison of the Chembio test vs. the EUA-approved Abbott Architect assay (see the “Discussion” section for additional detail).

During enrollment in our health care worker study, we sought to independently assess the performance characteristics of the Chembio test. First, for matrix analysis, 1 EDTA “lavender top” tube of blood from a venipuncture was obtained from 31 subjects with “reactive” COVID-19 antibodies and spun for collection of plasma. Testing was performed from these 3 matrices (finger stick, EDTA whole blood, and plasma). In addition, under a waiver of consent, we also received institutional review board (IRB) approval to obtain remnant serum and/or plasma from Stony Brook University Hospital’s clinical laboratory from patients with documented COVID-19 infection (PCR-positive nasopharyngeal swab) and patients with confirmed negative COVID-19 PCR testing.

### Statistical methods

Data from the Electronic Medical Record (EMR), interview responses, and antibody test results were collected and managed using REDCap software. All analyses were performed by the study’s data manager and statistician (co-author JR) using SAS Software© (Cary, NC).

For the primary analysis, all health care workers were categorized into three groups: (1) those who reported having *no* cold or flu-like symptoms experienced between 15 February 2020 and the day of antibody testing, (2) those who reported having cold or flu-like symptoms since 15 February 2020 but no positive PCR test (this included participants with negative PCR test results and those who had never been tested), and (3) those having at least one positive COVID-19 PCR test prior to having their antibodies tested regardless of symptoms. Differences in the proportions of antibody presence in the three groups were assessed using a chi-square test. Differences between the group pairs were also assessed (e.g., PCR+ group compared to the asymptomatic group). Chi-square tests were also used to assess differences in antibodies by profession and self-reported exposure to COVID-19 patients while at work. Binomial (Clopper-Pearson) exact 95% confidence intervals are reported with all antibody-positive results as a measure to capture uncertainty. The performance characteristics of the antibody test were calculated from the remnant serum and/or plasma, all of which had a known COVID-19 PCR+ or PCR test. Multiple remnant serum and/or plasma samples were collected, and seroconversion was plotted over time for PCR+ patients. Finally, for the matrix analysis, IgG antibody levels obtained from the finger stick were regressed on the IgG levels obtained from whole blood and plasma.

## Results

Overall, 474 health care workers were enrolled including 102 front-line physicians (intensivists, emergency medicine, anesthesiologists, surgeons); 91 other physicians; 135 nurses; 134 other clinical staff, e.g., respiratory therapists; and 12 other (non-clinical) hospital staff. The prevalence of IgM or IgG antibodies to SARS-CoV-2 was 16.9% (95% CI 13.6–20.6) (80/474) measured 25.5 [IQR 17.0–38.0] days after the onset of symptoms if applicable (Table 1).

**Table 1:** COVID-19 antibody prevalence among New York health care workers

Characteristic	Total		Negative for antibodies (IgG and IgM)		Positive for antibodies (IgG or IgM)		
**Total**	474		394	83.1%	80	16.9%	(13.6%, 20.6%)
**Sex—**no. (%)							
Male	182	38.4%	156	85.7%	26	14.3%	(9.6%, 20.2%)
Female	292	61.6%	238	81.5%	54	18.5%	(14.2%, 23.4%)
**Age**—mean (Std)	42.0	11.30	41.9	11.3	42.4	11.5	N/A
**Race**—no. (%)							
Non-Hispanic White	339	71.5%	286	84.4%	53	15.6%	(11.9%, 20.0%)
Hispanic or Latino	31	6.5%	24	77.4%	7	22.6%	(9.6%, 41.1%)
African American/Black	14	3.0%	13	92.9%	1	7.1%	(0.2%, 33.9%)
Asian	87	18.4%	68	78.2%	19	21.8%	(13.7%, 32.0%)
Others/unknown	3	0.6%	3	100.0%	0	0.0%	N/A
**Had a PCR test**—no. (%)	65	13.7%	48	73.8%	17	26.2%	(16.0%, 38.5%)
# Days from first PCR test to antibody test (must have occurred before the antibody test)—median, IQR	15	[11, 22]	14	[9, 20]	17	[13.0, 24.0]	N/A
**Symptom groups**—no. (%)							
Asymptomatic since 15 February 2020	244	51.5%	214	87.7%	30	12.3%	(8.5%, 17.2%)
Symptomatic, but no +PCR test prior to our AB samples	217	45.8%	178	82.0%	39	18.0%	(13.1%, 23.7%)
PCR+ test prior to our AB samples	13	2.7%	2	15.4%	11	84.6%	(54.6%, 98.1%)
**# Days from symptom onset to first Ab test** (*n* = 230) —median (IQR)	25.5	[17.0, 38.0]	24	[15.5, 39.0]	29.5	[21.0, 38.0]	N/A

Binomial exact 95% confidence intervals for proportions of positive antibodies are located in the right-most column of the table. Percentages for the total health care workers are presented as a proportion of the total (column), whereas percentages for the antibody groups are presented as proportions of the subgroup (row) *Std* standard deviation, *PCR* Polymerase chain reaction, *IQR* Interquartile range, *Ab test* Chembio COVID-19 antibody test

**Fig. 1:**
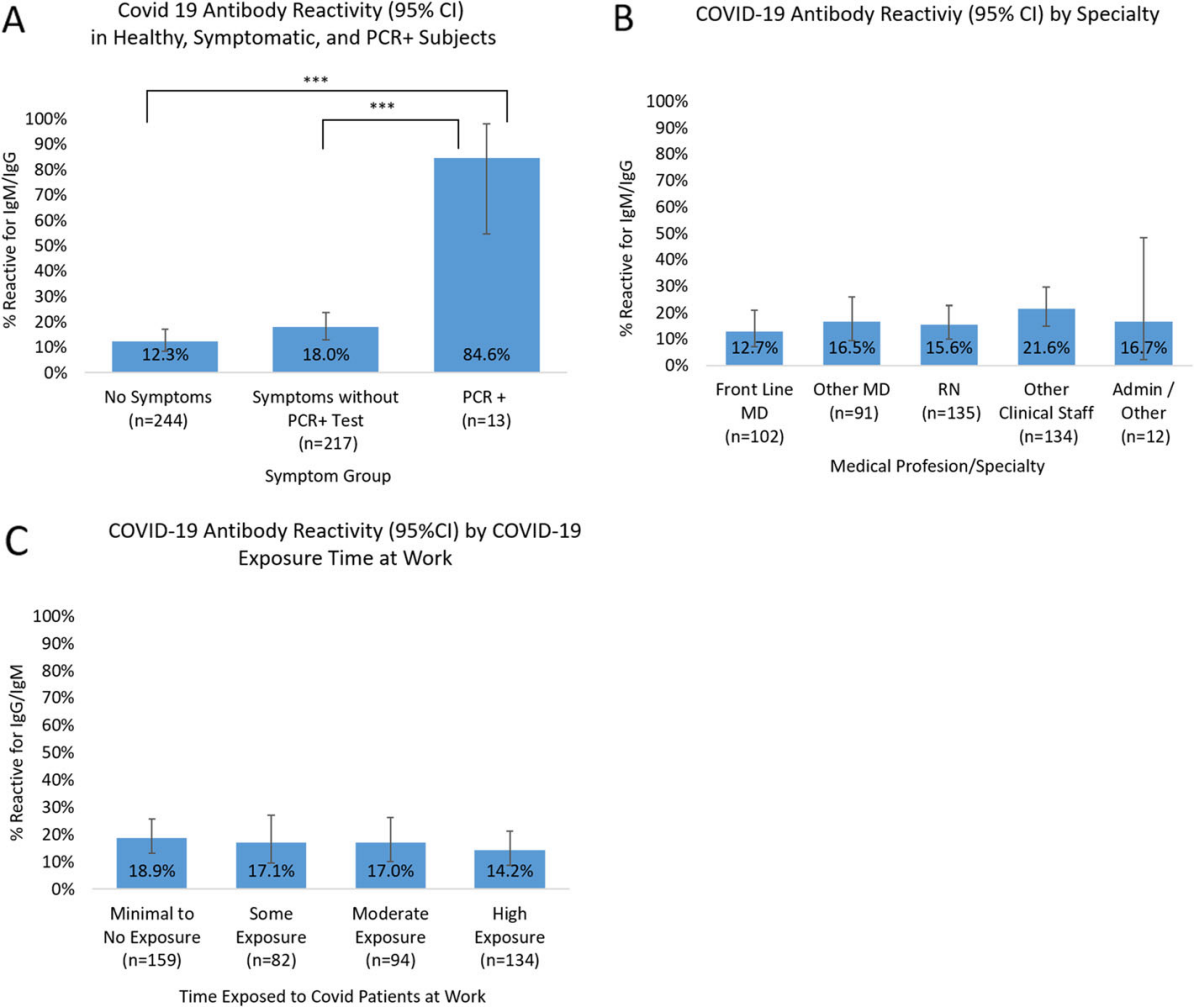
Prevalence of IgM and IgG antibodies to SARS-CoV-2 in health care workers (HCW). All panels show the proportion of prevalence plus binomial exact 95% confidence intervals (CI) as error bars. **a** Self-reported symptoms of illness after 15 February 2020, and responses were broken into 3 groups: no symptoms, symptoms without a PCR-positive test (this includes participants with a negative PCR test and those who had never been COVID-19 PCR tested), and PCR-positive test regardless of symptoms. The proportion of positive antibodies in the PCR + group was significantly higher than both the healthy group (84.6% vs. 12.3%, chi-square *p* < 0.001) and the symptoms group (84.6% vs. 18.0%, chi-square *p* < 0.001). The presence of antibodies in the healthy and symptomatic groups was marginally different (12.3% vs. 18.0%, chi-square *p* = 0.09). **b** HCW self-reported exposure to COVID-19 patients at work showed no statistically significant difference in proportions of COVID-19 antibodies among the different exposure groups. Those in which exposure assessments were not applicable (*n* = 5) were excluded. **c** Health care workers tested for COVID-19 antibodies reported by medical profession: front-line MDs included Emergency Medicine and Intensive Care Unit doctors, anesthesiologists, and surgeons. Other clinical staff included nurse practitioners, physician assistants, respiratory and radiology technicians, and transport staff. Administrative/other included other (non-clinical) hospital staff. There were no statistically significant differences in proportions of positive antibodies between the professional groups

Self-reported symptoms of illness and responses were broken into 3 groups (Fig. 1a): no symptoms since 15 February 2020, symptoms without a PCR-positive test (including participants with negative COVID-19 PCR test results and those who had never been tested), and PCR-positive test regardless of symptoms. The proportion of positive antibodies in the PCR + group was significantly higher than both the healthy group (84.6% [95% CI 54.6–98.1] vs. 12.3% [95% CI 8.5–17.2], *p* < 0.001) and the symptoms group (84.6% vs. 18.0% [13.1–23.7], *p* < 0.001). HCW self-reported exposure to COVID-19 patients at work showed no difference in proportions of COVID-19 antibodies among the different exposure groups (Fig. 1b) and among the different medical professionals (Fig. 1c).

**Fig. 2:**
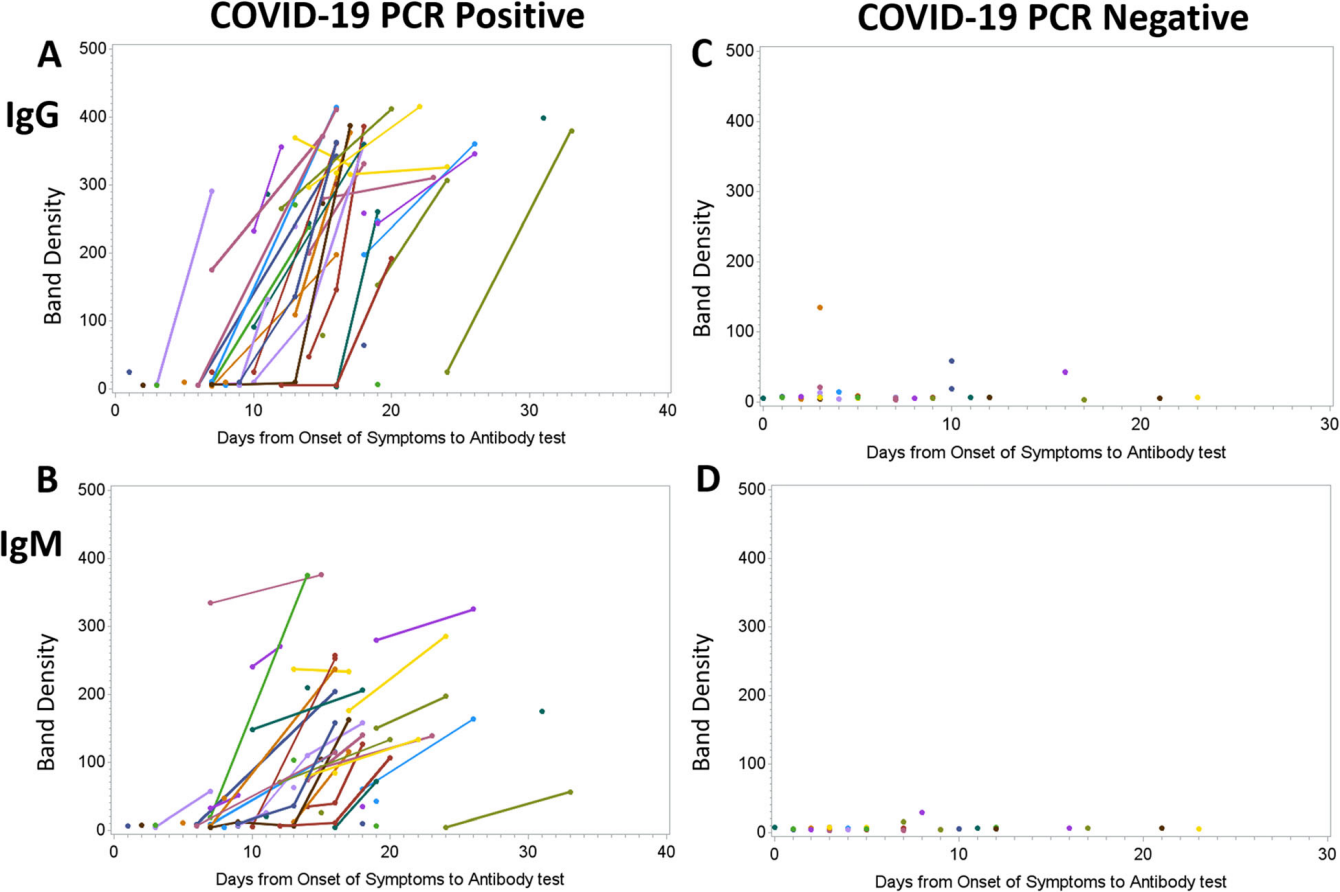
Seroconversion IgM and IgG change over time among PCR-confirmed + and − COVID-19 patients. Antibody levels (measured in reflectance light units) were measured in remnant-sample blood from patients with confirmed COVID-19 PCR-positive (**a**, **b**) and PCR-negative (**c**, **d**) test results

Performance characteristics (Fig. 2) of the antibody test from our laboratory remnant samples revealed a sensitivity of 84.3% and specificity of 88.9%. Finally, in 31 HCW research subjects, an analysis comparing finger stick, EDTA whole blood, and plasma antibody levels showed these to be highly correlated (Supplemental Figure 1).

## Discussion

In this single-center cross-sectional study of 474 health care workers, despite an increased exposure to COVID-19 patients, the prevalence of antibodies in health care workers (16.9%) was similar to that from initial reports of the general population in New York and Long Island (Morosky, 2020; Rosenberg et al., 2020). It is also similar to a published health care worker cohort from neighboring Nassau County, adjacent to New York City (Moscola et al., 2020). This is a single “snapshot” during the “surge” in New York when COVID-19 patients comprised over 2/3rd of our Hospital’s inpatients, with 120 of these patients being intubated.

We observed no significant difference in the prevalence of antibody reactivity for four levels of self-reported degree of exposure to COVID-19 patients, e.g., range “none” to “most of the time,” which is consistent with Moscola et al.’s study (Moscola et al., 2020). In their study involving 40,329 health care workers, the prevalence in those whose “job entailed working in a COVID-19 positive unit” was actually lower (12.3%) compared with those who did not work in a COVID-19 unit (16%). One interpretation of these results is that the use of personal protective equipment (PPE) and hand hygiene likely mitigates the risk of COVID-19 exposure health care workers face. There have been no shortages of personal protective equipment at our hospital. Surgical masks were required to be worn by all individuals within the hospital and alcohol-based hand sanitizing stations were densely positioned throughout the hospital. Additional personal protective equipment including N-95 masks, gloves, face shields, disposable gowns, and hospital laundered scrubs were provided for staff with patient contact. It is also possible that staff who interact closely with very sick COVID-19 patients, e.g., front-line nurses, may have heightened awareness and concern leading to better self-protection at work and in their communities, e.g., maintaining social distancing. Consistent with this postulated efficacy of personal protective equipment and hand hygiene, we found that antibody prevalence was similar among the different health care workers enrolled, e.g., nurses who perform more continuous direct patient care had similar prevalence to those with more supervisory less “hands-on” roles, e.g., physicians.

Of note, study participants who identified as Hispanic or Latino (22.6%) and Asian (21.8%) had higher rates of COVID-19 seropositivity than Non-Hispanic White (15.6%) or Black (7.1%) participants. This observation is consistent with results from Moscola et al.’s in which they observed lower rates of COVID-19 seropositivity in White subjects when compared to all other racial groups. It has been speculated that the higher incidence of COVID-19 in these racial groups may be in part related to socioeconomic factors and lower education status (Hawkins et al., 2020; Wiemers et al., 2020). As we did not ask our participants to report metrics of socioeconomic status, we are unable to draw conclusions regarding a connection between seropositivity and socioeconomic status. Interestingly, participants categorized as “other clinical staff” had higher rates of seropositivity (21.6%) compared to physicians (front line 12.7%, other MD 16.5%), nurses (15.6%), and administrative/non-clinical staff (16.7%). “Other clinical staff” included respiratory and radiology technicians, patient transportation staff, and nurse’s aides, professions with overall lower pay than nurses and physicians, but with opportunities nonetheless for COVID-19 exposure at work and in their communities. Providing adequate personal protective equipment and education to all hospital staff is essential to protect vital front-line workers who keep our hospitals running and communities healthy.

Our study has several limitations. Although serology testing was done in New York, the epicenter of the COVID-19 crisis, the study was a single center’s experience. As we asked health care workers to self-report exposure to COVID-19 patients, we cannot guarantee the accuracy of the relative time spent in these encounters. Like many published COVID-19 serology reports (Rosenberg et al., 2020; Moscola et al., 2020), we used a convenience sample, meaning individuals who thought they were infected with COVID-19 were potentially more likely to volunteer than if we had randomly sampled health care workers. Although FDA determined that Chembio’s DPP IgM/IgG assay was suboptimal in terms of reduced ability to detect antibodies in low titer specimens and lower specificity compared to other serology platforms, we believe our data are still valuable from a research perspective due to high internal validity and a scientifically sensible antibody target, the nucleocapsid viral protein, which has been used in other published cohort studies (Rosenberg et al., 2020; Moscola et al., 2020). The Chembio test’s EUA was revoked, in part, due to an FDA/National Cancer Institute evaluation utilizing a reference panel with low and high titer specimens. The Chembio IgG test had a sensitivity of 78.6% and a specificity of 91.2% which were below the FDA-recommended thresholds for sensitivity/PPA (≥ 90%) and specificity/NPA (≥ 93%). After the conclusion of our study, our hospital’s clinical laboratory, in response to the FDA’s action, and as part of its internal quality program, compared Chembio IgG test results against its other in-house COVID-19 antibody test, the EUA-approved Abbott Architect SARS-CoV-2 IgG assay, on 235 specimens. All of the Architect IgG-positive specimens were positive with the Chembio IgG test; however, most of these specimens had a relatively high concentration of antibodies based on the Architect IgG index values. Approximately 12% of the Architect-negative specimens were positive on the Chembio IgG test (negative percent agreement of 88%); most of the discordant positive Chembio tests had RLU values < 100 whereas most of the concordant Chembio positive tests had RLU values > 150. Since the Chembio assay was used to test all of the healthcare workers in our study, any limitations on the performance characteristics of the Chembio assay should not have affected the results across the different categories of health care workers.

Our study has several strengths. This “snapshot” focused on health care workers from a New York tertiary care hospital during the Spring 2020 surge when approximately 69% of patients in our hospital were COVID-19 PCR+ (56% of total) or a “patient under investigation” for COVID-19 (13% of total). Both our overall seroprevalence rate and the lack of correlations we observed relating to COVID-19 exposure and health care worker profession and seroprevalence are consistent with other published reports from across New York (Morosky, 2020; Rosenberg et al., 2020; Moscola et al., 2020). Our independent evaluation of the assay performance (Figs. 1a and Fig. 2) shows the test measures antibodies relevant to COVID-19 infection, even though the specificity of the assay may be suboptimal. The Chembio assay measures antibodies generated against the nucleocapsid (NP), a highly immunogenic viral structure, which is the target of many emergency use authorization serology assays, e.g., Abbott Architect, and was used in previously published seroprevalence studies (Rosenberg et al., 2020; Moscola et al., 2020). To assess the internal validity of the Chembio Assay, in a subset of health care worker participants, whole blood used for the antibody assay was collected from both a finger stick and a venipuncture. The IgG values of finger stick and venipuncture from individual participants were tightly correlated (Supplement Figure 1) further supporting the strength of our data set. Our study’s substantial internal validity enabled us to confidently draw comparisons between groups within the test cohort, e.g., high vs low exposure. In an attempt to survey large populations, some groups have pooled results from multiple serology platforms all with varying metrics and antibody targets. Our testing was done using a single assay reducing this variability considerably.

In summary, despite exposure to COVID-19 patients, the prevalence of antibodies in our HCW was similar to what has been reported for the general population of New York State (14%) and for another New York HCW cohort (13.7%). Health care workers with higher exposure rates were not more likely to have been infected with COVID-19. Therefore, these data suggest that infection of HCW may result from exposure in the community rather than at work.

## Supplementary Information

**Additional file 1: Supplement Figure 1** Finger stick IgG antibody levels correlate tightly with antibody levels of EDTA peripheral blood and plasma. Figures show correlations of IgG values from different matrices. Table shows percent positive (IgM or IgG), and median IQR for IgM and IgG from each of the 3 matrices obtained from 31 subjects

## Data Availability

Please contact the author for data requests.

## Abbreviations

HCW: Health care workers
PPE: Personal protective equipment
IRB: Institutional review board
EUA: Emergency use authorization
EMR: Electronic medical record
NP: Nucleocapsid protein
PCR: Polymerase chain reaction
PUI: Patient of interest
RLU: Reflectance light units
