# Supplementary material for: Prevalence of IgM and IgG antibodies to SARS-CoV-2 in health care workers at a tertiary care New York hospital during the Spring COVID-19 surge
Source: Perioper Med (Lond). 2021 Mar 2;10:7. doi: 10.1186/s13741-021-00177-5 (PMC7920632; doi:10.1186/s13741-021-00177-5)

Supplemental Figure 1 Finger stick IgG antibody levels correlate tightly with antibody levels of EDTA whole blood (A) and plasma (B)

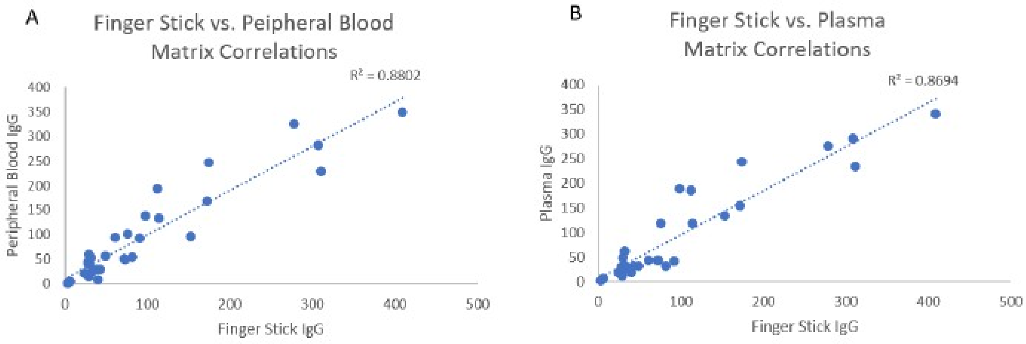

Supplement: Supplementary file 1 [file 13741_2021_177_MOESM1_ESM.docx]
